# Differences in Pop Levels between Conventional and Omega-3 Fatty Acid-Enriched Milk and Dairy Products

**DOI:** 10.5402/2011/541694

**Published:** 2011-07-10

**Authors:** Cristiana Guerranti, Silvano Ettore Focardi

**Affiliations:** Department of Environmental Sciences “G. Sarfatti”, University of Siena, Via Mattioli 4, 53100 Siena, Italy

## Abstract

Conventional and omega-3 fatty acid-enriched milk and cheese were analyzed for persistent organic pollutants (POPs). Omega-3-enriched products are usually supplemented with fish oil which is potentially contaminated. All classes of the considered POPs (PCBs, DDT, HCB, PBDEs, and PCDD/Fs) were found in the samples, with average concentrations higher in omega-3-enriched products than in conventional ones. For PCBs, DDT, and HCB, differences were statistically significant and, therefore, cannot be ascribed to normal variability. With regard to all classes of compounds, the highest levels in individual samples were always found in omega-3 products, in line with the hypothesis that these foods are potentially more contaminated than conventional ones.

## 1. Introduction

Persistent organic pollutants (POPs) are a large class of organic contaminants that pose potential risks for human health [1]. Since they are highly lipophilic and resist transformation, they bioaccumulate in animal and human adipose tissue [2]. Food is considered the main source of nonoccupational human exposure to most organohalogen compounds; indeed, foodstuffs of animal origin, especially meat, dairy, and fish products, contribute more than 90% of the human body burden [3]. Moderate to high levels of POPs are normally encountered in milk and dairy products because lactating animals eliminate lipophilic organohalogen compounds from their fatty tissue through milk production [2]. Dairy products are usually high in fat and may, therefore, contain large quantities of lipophilic contaminants.

Consumption of milk and dairy products supplemented with omega-3 fatty acids is advertised as beneficial for health. However, the purported benefits of these products, borderline between food and drugs, are often based more on ideological and emotional factors than on complete scientific research. Omega-3-enriched products may not be as beneficial as they are claimed to be. Enriched products are usually supplemented with fish oil, which besides being a good source of high-quality proteins, minerals, vitamins, and polyunsaturated omega-3 fatty acids, is extracted from fish, products potentially very contaminated by compounds such as pesticides and dioxins [3].

In this study we analyzed samples of conventional and omega-3-enriched milk and cheese for POPs levels.

## 2. Materials and Methods

Analysis of two chlorinated pesticides (HCB and DDTs), 19 PBDE, seven PCDDs, ten PCDFs, and 57 PCBs was performed on 30 pools of milk and cheese acquired from three supermarkets in central Italy. The pools consisted of small portions of the different foods purchased, typically four or five samples, often of the same product differently packaged. They were placed in containers decontaminated with acetone and n-hexane and stored at −20°C until analysis.

PCB congeners (IUPAC numbers CB18, CB22, CB26, CB28, CB31, CB33, CB40, CB42, CB44, CB49, CB52, CB77, CB81, CB95, CB99, CB101, CB105, CB110, CB114, CB118, CB123, CB126, CB128, CB134, CB136, CB137, CB138, CB141, CB146, CB149, CB151, CB153, CB156, CB157, CB158 CB167, CB169, CB170, CB171, CB172, CB174, CB176, CB177, CB178, CB180, CB183, CB185, CB187, CB189, CB194, CB195, CB196, CB199, CB201, CB202, CB205, CB206, and CB207) and PBDEs (IUPAC numbers BDE3, BDE7, BDE5, BDE17, BDE28, BDE49, BDE71, BDE47, BDE66, BDE77, BDE100, BDE119, BDE99, BDE85, BDE126, BDE154, BDE153, BDE138, and BDE156), were selected on the basis of their presence in commercial mixtures, the environment, and body tissues. Throughout the paper PCBs and PBDEs are represented by their IUPAC numbers. The seventeen PCDD and PCDF congeners analyzed were those substituted in positions 2, 3, 7, and 8.

### 2.1. Chemical Analysis

Analysis of samples was done following the method proposed by Corsolini et al. [4] with some modifications. To extract and purify organohalogen compounds from solid samples, the products were reduced to small pieces and homogenized with a *Buchi Mixer *at the time of analysis. An aliquot of fresh sample (about 10 g) was dehydrated with anhydrous sodium sulphate, spiked with internal standard containing PCB 30, PCB 209, and PBDE 139 ^13^C (supplied by Supelco and Cambridge Isotope Laboratories, respectively) and a mixture of PCDD/DF ^13^C (Cambridge Isotope Laboratories) as envisaged by EPA quantification method 1613, and digested in a Soxhlet apparatus with dichloromethane and n-hexane (3 : 1) at 120°C for 16 h. An aliquot of the extract (about 10%) was used to determine lipid content by gravimetry. The remaining aliquot was concentrated to 10 mL in a rotary evaporator and eluted with n-hexane on a chromatograph column packed with 100–200 mesh nonactivated silica gel and acidified silica gel (40% H_2_SO_4_, 60% silica gel). The eluate was concentrated in a rotary evaporator and placed in vials for gas chromatography. If the sample was visibly still fatty after silica-gel column purification, it was cleaned up with sulphuric acid.

Milk samples were freeze-dried before Soxhlet extraction and after silica column chromatography, and if they were still too fatty to concentrate, they were washed with a small volume of sulphuric acid in the test tube. 

The resulting extracts were used for the determination of chlorinated pesticides, PBDE, and total PCBs.

A Perkin Elmer Autosystem gas chromatograph with Ni^63^ electron capture detector and a Supelco SBP-5, bound phase fused silica capillary column (length 30 m, i.d. 0.2 mm, film thickness 125 *μ*m) was used for PCB and pesticide analyses. The carrier gas was helium at 15.5 psi; the scavenger gas was argon/methane (95/5). The split-splitless injector was used in splitless mode at a temperature of 270°C. Detector temperature was 300°C. The oven programme was 120°C for 1 min, to 180°C at 25°C/min, to 280°C at 5°C/min, and hold for 10 min. Contaminants were determined by comparing the results with those of external standards of known concentration and composition and at least 99% purity:

- Aroclor 1260 (Supelco Inc., US EPA-certified),
- mixtures of various mono-ortho-PCB congeners (Dr. Ehrenstorfer GmbH),
- a mixture of HCB and DDT isomers and congeners (Dr. Ehrenstorfer GmbH).

PBDEs were identified and quantified using a Trace GC 2000/Polaris ion trap mass spectrometer equipped with an *AS2000* autosampler (ThermoFinnigan) and a Restek *Rtx-5MS* capillary column (30 m, i.d. 0.25 mm, film thickness 0.25 *μ*m). A 2 *μ*L aliquot of sample in isooctane was injected in splitless mode with helium as carrier gas. Injector temperature was 275°C. The ramp program was 80°C for 2 min, to 200°C at 25°C/min, to 300°C at 4°C/min, and hold 10 min. Excitation voltages were 4.75 V for tri- and tetra-BDEs, 4.60 V for penta-BDEs, and 4.70 V for hexa-BDEs. The internal standard was PCB14 ^13^C in isooctane (Cambridge Isotope Laboratories), and the PBDE standard solution for calibration was from Wellington Laboratories Inc. 

To analyse non-ortho-PCBs 77, 81, 126, and 169, furans, and dioxins, the rest of the extract was concentrated and run on a silica-impregnated activated carbon chromatograph column (Wako). The column (i.d. 10 mm) was packed with sodium sulphate and 1 g activated carbon. The first elution was with hexane, followed by elution of the sample extract, then another elution with hexane. The eluate of these phases was discarded. The eluate of a final elution with toluene was collected and concentrated in a rotary evaporator for analytical determination.

Non-ortho PCBs were analysed by mass spectrometry using a spectrometer with Finnigan *Polaris Q* ion trap detector coupled with a *Trace GC Ultra* chromatograph with *AI3000* automatic sampler (ThermoFinnigan) and a Restek *Rtx-5MS* capillary column (30 m × 0.25 mm, i.d. 0.5 *μ*m). To determine coplanar PCBs, 2 *μ*L of each sample extract was injected in splitless mode at 250°C. Oven temperature was 110°C for 1 min, to 220°C at 20°C/min, hold 2 min, to 300°C at 10°C/min, and hold 5 min. Molecular ionisation was obtained by electronic impact, and scans were performed in MS/MS mode with an excitation voltage of 6.60 V. A mixture of four non-ortho PCBs at five concentrations supplied by AccuStandard Inc. was used to plot the calibration curve.

PCDD and PCDF were determined by EPA method 1613 according to the European Community directive 2002/69/CE. 2 *μ*L of each sample extract, brought to final volume with the recovery standard specified by EPA 1613, was injected in splitless mode at 250°C. Oven temperature was 110°C for 1 min, to 220°C at 20°C/min, hold 2 min, to 300°C at 10°C/min, and hold 5 min. Molecular ionisation was obtained by electronic impact, and scans were performed in MS/MS mode. Excitation voltages were 4.40 V for tetra-, penta-, and hexa-CDD, 4.50 V for hepta-CDD, 4.75 V for tetra-CDF, 4.90 V for octa-CDD, 5.05 V for penta-CDF, 5.30 V for hexa-CDF, and 5.50 V for hepta- and octa-CDF. A mixture of PCDD/F at five concentrations from AccuStandard Inc. was used for the calibration curve.

The limits of detection (LOD) of the compounds were the concentrations detected in blanks +3 SD and on the average were 0.01 ng/g for PCB congeners, HCB, and DDTs, 0.02 pg/g for PBDE congeners, and 0.5 pg/g for PCDD/F congeners.

Results (lipid basis) of pairs of food groups were compared using the Mann-Whitney-Wilcoxon nonparametric test. Statistical analysis was run with the programmes Microsoft *Excel 2003 *(Microsoft Corporation) and *Statistica 7.1 *(StatSoft Inc.), both for *Windows XP. *Results are given on a wet weight basis (w.w.) and on a lipid basis (l.b.). For calculations, concentrations below the LOD were considered as zero.

### 2.2. Quality Control

The accuracy of the analytical procedure was determined by analysis of certified materials from the National Institute of Standards and Technology, US Department of Commerce (Gaithersburg, Md, USA), the National Council of Canada, Institute for National Measurement Standards, and the Community Bureau of Reference (BCR). The following parameters/compounds were determined in the following materials: HCB and total DDTs in pig fat (NIST), fat content in homogenized meat (NIST); pesticides and total PCBs in cod liver oil (NIST), PCDD/F and PCBs in carp muscle (CARP-1, NRC), total PCBs and PBDE-47 in pig fat (ERM-IRMM), and total PCBs (BCR 450), PCDD, and PCDF (BCR RM 534) and pesticides (BCR 187) in powdered milk. Recovery was more than 88% in all cases. Recoveries were also evaluated by the method of additions at the time of extraction, using homogeneous replicates and were always greater than 90%. A blank was analysed with every analysis series (five samples).

## 3. Results and Discussion

The samples had the following fat contents:

(i) semiskimmed milk 2.8–3.1%,

(ii) “stracchino” (Italian soft cow cheese) 25.3–25.8%,

(iii) “pecorino” (Italian sheep cheese) 23.5–24.0%,

(iv) “ricotta” (Italian low-fat cheese) 15.8–16.1%.

All classes of contaminants analysed were found in the samples (Tables 1 and 2). Means and standard deviations were calculated for each group of foods.

**Figure 1:**
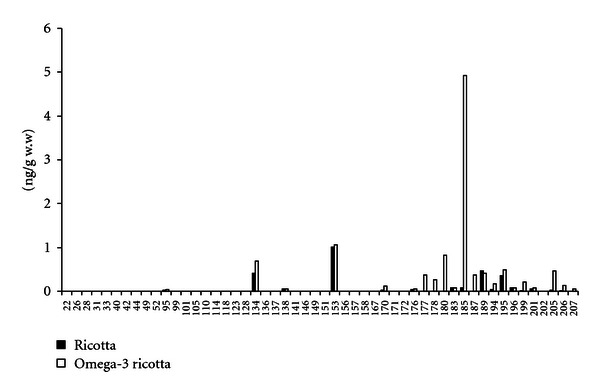
PCBs congener fingerprints in conventional samples of ricotta and omega-3-enriched ricotta.

In almost all pools, the prevalent PCB congener was PCB-153, followed by PCB-134 and PCB-189. Figure 1 shows an example of fingerprints of PCB congeners in conventional samples of ricotta and omega-3 ricotta: for the former, the prevalence ranking was the same as in most samples of conventional dairy products, whereas in the latter, high concentrations of PCB-185 and many other medium to highly chlorinated congeners, absent in conventional milk and dairy products, were found. 

**Figure 2:**
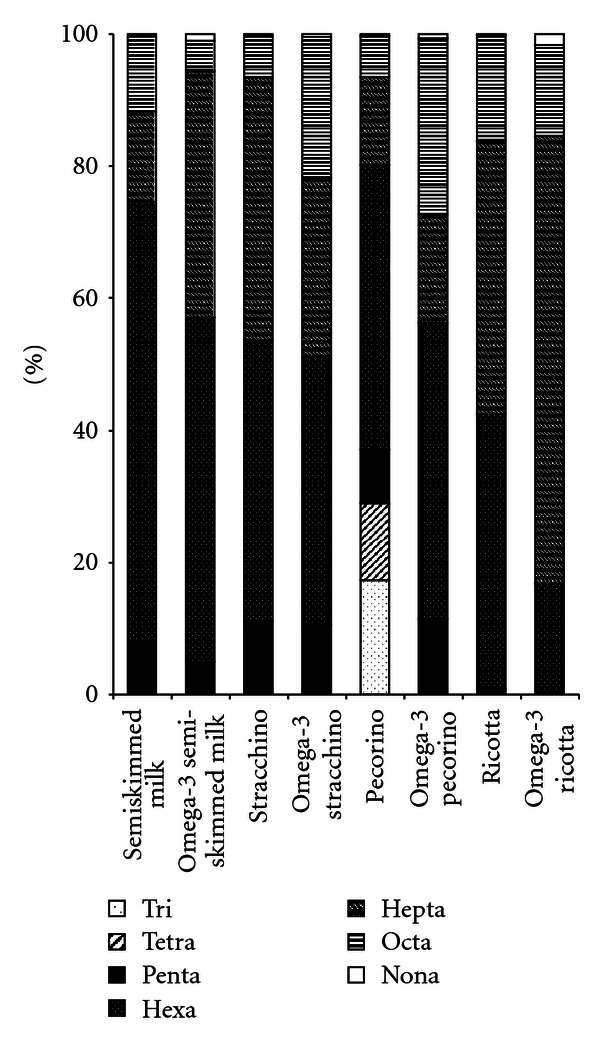
PCB isomer composition in the various dairy foods.

Among PCBs there was a prevalence of hexa- followed by hepta- and octachlorobiphenyls (39%, 38%, and 11%, respectively, of mean total PCBs in all products). Trichlorobiphenyls were the lowest (1%).  Figure 2 shows the PCB isomer composition in the various dairy foods. 

The most abundant DDT compound was *pp*′-DDE (95%), followed by *op*′-DDE (4%), *op*′-DDD, and *pp*′-DDT (<1%). The latter three compounds were found in a limited number of pools of samples. The prevalence of *pp*′-DDE, a metabolite of DDT degradation found in the environment [8], suggests direct ingestion and/or its accumulation in food chains, rather than exposure of the animal producing the milk to the compound [9].

PBDEs were found in cheese but never in milk. Tetra- and pentabromide congeners such as PBDE-47 and PBDE-99 were prevalent. 

Among non-ortho-PCBs, congener 126 was prevalent, followed by 77. Congeners 81 and 169 were never found. Among PCDFs and PDCCs, the latter was preponderant in all samples analysed.

TEQs were calculated on the basis of concentrations of PCDD, PCDF, and certain PCB congeners [10, 11]; none of the samples exceeded the WHO-TEQ 1998 safety limit adopted by the European Union (4 pg/g lipid basis, l.b.).

The HCB levels encountered in the products can be compared with those reported by Leoni and colleagues [12] who found a range of 0.40–9.00 ng/g wet weight (w.w.), mean 3.15 ng/g w.w. in cheese, which is higher than our values (mean 1.67 ng/g w.w. in omega-3 and 0.14 ng/g w.w. in conventional products). This difference could be due to a reduction in levels of this contaminant since it was banned. Compared to our results, Kannan et al. [13] reported a lower mean concentration of HCB in cheese from Moresby (Papua New Guinea): 0.43 ng/g w.w.

With regard to milk, our results can be compared to those of Kiviranta and colleagues [5], Schmid et al. [7], and Falcò et al. [6] for Finnish, Swiss, and Spanish milk samples (Table 3).

Application of the Mann-Whitney-Wilcoxon test to differences between values obtained for the pairs of foods by descriptive statistics, which showed that omega-3-enriched foods were more contaminated than conventional ones, confirmed this difference for *∑*PCBs, HCB, and *∑*DDT (W = 90, *P* = 0.0103, W = 92, *P* = 0.0052, and W = 92, *P* = 0.0052, resp.).

## 4. Conclusions

Mean concentrations of the compounds analysed were clearly higher in omega-3-enriched products than in conventional ones. Specifically, differences in mean concentrations of PCBs, DDT, and HCB, all compounds, banned decades ago in Italy, were statistically significant, showing that the different levels of contamination were not due to normal variability of results but to actual addition of contaminants through addition of fish oil to milk and cheese.

The finding of higher levels of all contaminants in individual samples of omega-3-enriched foods sustains the hypothesis that these products may be more contaminated by lipophilic pollutants than traditional products. It would, therefore, be worthwhile doing a cost-benefit analysis of omega-3 dietary supplements which are certainly beneficial for the cardiovascular system but may simultaneously expose consumers to dangerous environmental contaminants.

## Figures and Tables

**Table 1:** Contaminant concentrations in milk and cheese: summary of analytical results (*lower bound,* wet weight basis). ND indicates that all values in the group were below the LOD and were, therefore, considered equal to zero; M: mean; SD: standard deviation.

Food group		*∑*PCBs ng/g w.w.	HCB ng/g w.w.	*∑*DDTs ng/g w.w.	*∑*PBDEs ng/g w.w.	*∑*non-ortho-PCBs pg/g w.w.	*∑*PCDD/Fs pg/g w.w.	WHO-TEQ 1998 pg/g w.w.	WHO-TEQ 2006 pg/g w.w.
Conventional semiskimmed milk	M	1.15	0.53	0.50	ND	ND	ND	ND	ND
	SD	0.03	0.01	0.16	—	—	—	—	—

omega-3 semiskimmed milk	M	10.31	0.5	1.08	ND	ND	ND	ND	ND
	SD	1.24	0.2	0.21	—	—	—	—	—

Conventional “stracchino”	M	5.19	0.22	0.22	10.94	ND	ND	ND	ND
	SD	0.7	0.18	0.18	11.05	—	—	—	—

omega-3 “stracchino”	M	1.85	1.76	3.84	10.51	ND	ND	ND	ND
	SD	0.27	0.6	0.46	11.34	—	—	—	—

Conventional “pecorino”	M	2.02	ND	ND	13.82	ND	ND	ND	ND
	SD	0.01	0	0	9.01	—	—	—	—

omega-3 “pecorino”	M	6.97	2.94	5.87	11.41	2.36	1.56	0.13	0.07
	SD	0.72	0.22	1.40	0.47	7.57	1.62	0.23	0.12

Conventional “ricotta”	M	2.75	0.21	0.07	1.32	1.53	1.06	0.02	0.01
	SD	0.07	0.14	0.04	0.38	2.15	0.05	—	—

omega-3 “ricotta”	M	10.98	0.3	0.12	3.31	3.79	2.92	0.02	0.01
	SD	0.54	0.14	0.11	0.34	0.20	0.59	0.06	0.02

**Table 2:** Contaminant concentrations in milk and cheese: summary of analytical results (*lower bound,* lipid basis). ND indicates that all values in the group were below the LOD and were, therefore, considered equal to zero; M: mean; SD: standard deviation.

Food group		*∑*PCBs ng/g l.b.	HCB ng/g l.b.	*∑*DDTs ng/g l.b.	*∑*PBDEs ng/g l.b.	*∑*non-ortho-PCBs pg/g l.b.	*∑*PCDD/Fs pg/g l.b.	WHO-TEQ 1998 pg/g l.b.	WHO-TEQ 2006 pg/g l.b.
Conventional semiskimmed milk	M	63.71	29.24	27.68	ND	ND	ND	ND	ND
	SD	1.60	0.51	8.70	—	—	—	—	—

omega-3 semiskimmed milk	M	573.04	27.68	59.75	ND	ND	ND	ND	ND
	SD	68.94	11.22	11.59	—	—	—	—	—

Conventional “stracchino”	M	20.73	0.89	0.87	63.55	ND	ND	ND	ND
	SD	2.79	0.71	0.70	89.87	—	—	—	—

omega-3 “stracchino”	M	7.37	7.02	15.28	65.76	ND	ND	ND	ND
	SD	1.07	2.37	1.82	93.00	—	—	—	—

Conventional “pecorino”	M	8.31	ND	ND	89.78	ND	ND	ND	ND
	SD	0.04	—	—	126. 7	—	—	—	—

omega-3 “pecorino”	M	28.70	12.09	24.17	67.53	8.32	10.29	0.53	0.30
	SD	2.95	0.91	5.76	1.92	26.58	5.87	0.90	0.94

Conventional “ricotta”	M	16.26	1.24	0.42	7.76	7.54	8.43	0.17	0.10
	SD	0.39	0.84	0.22	2.25	16.34	0.29	0.02	0.01

omega-3 “ricotta”	M	66.13	1.78	0.75	19.94	24.86	24.98	0.16	0.09
	SD	3.24	0.83	0.64	2.04	0.98	3.98	0.57	0.13

**Table 3:** Mean concentrations of contaminants in milk reported by Kiviranta et al. [5] and Falcò et al. [6] on a wet weight basis, and mean ± standard deviation by Schmid et al. [7] on a lipid basis. Data in ng/g for total PCBs, PBDE and HCB and in pg/g for the other contaminants/parameters.

Kiviranta et al. [5]	Falcò et al. [6]	Schmid et al. [7]
***∑***PCBs 2.6	HCB 0.01	WHO-TEQ 1998 0.59 ± 0.22
***∑***non-ortho-PCBs 2.1	***∑***PCBs 1.28	
***∑***PCDD/Fs 2.5	***∑***PBDEs 0.04	
	WHO-TEQ 1998 PCDD/Fs 0.22	

## Abbreviations

DDT: dichlorodiphenyltrichloroethane
HCB: hexachlorobenzene
PBDEs: polybrominated diphenyl ethers
PCBs: polychlorinated biphenyls
PCDD/Fs: polychlorinated dibenzodioxins/furans.
